# Therapeutic Effects of Chinese Herbal Formula (PTQX) on NC/Nga Mice with Atopic Dermatitis-Like Skin Lesions

**DOI:** 10.1155/2019/8359252

**Published:** 2019-12-07

**Authors:** Fenggen Yan, Jing Zhang, Xiong Li, Xiumei Mo, Junfeng Liu, Siqi Ye, Ying Lin, Xiaohui Qiu, Simon Mingyuen Lee, Dacan Chen

**Affiliations:** ^1^Department of Dermatology, The Second Affiliated Hospital of Guangzhou University of Chinese Medicine, Guangdong Provincial Hospital of Chinese Medicine, Guangdong Provincial Key Laboratory of Chinese Medicine for Prevention and Treatment of Refractory Chronic Diseases, Guangzhou, Guangdong, China; ^2^State Key Laboratory of Quality Research in Chinese Medicine and Institute of Chinese Medical Sciences, University of Macau, Macau, China

## Abstract

Atopic dermatitis (AD), also known as atopic eczema, is a chronic pruritic inflammatory skin disease. The available systemic therapies for atopic dermatitis are inadequate. *Objective*. This study aimed to evaluate the effects of the Chinese herbal formula Pei Tu Qing Xin (PTQX) on dermatitis severity and ear swelling, immunomodulation, and the infiltration of mast cells in a mouse model of 1-chloro-2,4-dinitrobenzene- (DNCB-) induced AD. *Methods*. AD-like symptoms were induced by DNCB in NC/Nga mice. Skin lesions, dermatitis, ear swelling, and scratching behaviour were evaluated. Changes in the T-helper type 1 (Th1), Th2, Th17, and regulatory T (Treg) subtypes and immunoregulation in the spleen and lymph nodes were detected by flow cytometry. *Results*. Histopathological and immunohistochemical analyses demonstrated that PTQX decreased the DNCB-mediated induction of mast cells and infiltration of inflammatory cells in the ear and dorsal skin. PTQX also reduced the DNCB-induced increase in the serum immunoglobulin E level, pruritus, and dermatitis (red, flaky areas) on the dorsal skin. Furthermore, PTQX regulated the balance between the populations of Th1, Th2, Th17, and Treg cells (particularly the latter two) in the lymph nodes. *Conclusions*. Our results suggest that the Chinese herbal formula PTQX can alleviate symptoms of AD, such as epithelial damage, redness, swelling, and pruritus, and potentially be used to treat this condition.

## 1. Introduction

Atopic dermatitis (AD) is a chronic, highly treatment-refractory form of itchy inflammatory skin dermatosis that affects both children and adults. It is among the most common chronic skin diseases, affecting up to 25% of children and 2-3% of adults in most countries worldwide [1]. Clinically, AD is a chronic form of pruritic inflammatory skin disease characterised by red, burning, dry, swollen, and cracked skin. The pathogenesis of AD is complex and involves multiple contributors such as skin barrier defects, infections, immunological factors (T-helper (Th) cells, inflammatory cells, immunoglobulin E, cytokines, and chemokines), susceptible genes (filaggrin genes), and environmental interactions (food and aeroallergens and seasonal and climatic changes) [2–4]. Currently, no treatment can effectively cure or control the recurrence of AD. No standardised international guidelines have been set for AD, and the internally and topically administered hormone or immune inhibitors and antihistamines used in traditional Western medicine elicit side effects and are not ideal. Therefore, alternative medicinal treatments for AD, including traditional Chinese medicine (TCM), have elicited broad and increasing interest in recent years [5, 6].

TCM has been used clinically for thousands of years, including the routine management and treatment of AD, and its efficacy has been proven through scientific investigation [7]. A recent expert consensus recommended the TCM formula Pei Tu Qing Xin (PTQX) as a prescribed treatment for AD [8]. Previously, our team reported an outcome assessor-blinded, placebo-controlled, 3-arm randomised clinical trial (RCT) conducted to evaluate the efficacy and safety of PTQX for the treatment of AD. In our study, PTQX effectively relieved the symptoms of AD and improved the self-assessed quality of life (QOL) in patients with moderate to severe AD, with no reported severe adverse events [9]. Furthermore, a study protocol for a RCT intended to test the efficacy and safety of oral PTQX in children aged 6–16 years with moderate-to-severe atopic eczema has been published in trials [10]. However, the molecular mechanisms underlying the effects of PTQX on AD remain unclear.

Our previous study indicated that PTQX considerably relieved the symptom of pruritus in a guinea pig model by reducing the release of endogenous and exogenous histamine [11]. The anti-inflammatory effects of PTQX were assessed in 3 different animal models: xylene-induced oedema in the mouse ear, cotton pellet granuloma formation, and an acetic acid-induced increase in abdominal cavity capillary permeability [12]. A further investigation focused on the effects of PTQX on Th1 and Th2 cell differentiation in the spleens of C57BL/6 mice with induced AD. Compared with the model group, mice in the PTQX group exhibited reduced inflammation and regulation of the Th1/Th2 cell balance [13].

This study aimed to evaluate the therapeutic effects of PTQX on 1-chloro-2,4-dinitrobenzene- (DNCB-) induced AD-like symptoms in NC/Nga mice. We demonstrate here that PTQX can improve the symptoms of AD and reduce the inflammatory infiltration of CD4+ and CD8+ T cells into DNCB-induced skin lesions in these mice. We further evaluated the anti-inflammatory and antiallergic properties of PTQX *in vivo* and found that this treatment inhibited the total production of immunoglobulin E (IgE) as measured in serum, suppressed ear swelling, and significantly alleviated scratching behaviour. Furthermore, our experimental results demonstrate that PTQX inhibited the infiltration of mast and other inflammatory cells in skin lesions and regulated the Th/regulatory T (Treg) cell balance in lymphoid organs. Our findings suggest the potential therapeutic effects of the Chinese herbal formula PTQX against DNCB-induced AD-like skin lesions in NC/Nga mice.

## 2. Materials and Methods

### 2.1. Chemicals, Reagents, and Materials

High-performance liquid chromatography (HPLC) grade acetonitrile, methanol, and formic acid were obtained from Sigma-Aldrich (St. Louis, MO, USA). Ultrapure water was prepared using a Milli-Q water system (Millipore, Billerica, MA, USA). Chemical references, including liquiritin, liquirtigenin, isoliquiritin, isoliquiritigenin, and glycyrrhizin, were purchased from Chengdu Must Bio-Technology Co., Ltd. (Chengdu, China). Isoforsythiaside A, forsythoside A, and forsythin were obtained from Shanghai YuanYe Biotechnology Co., Ltd. (Shanghai, China). Chlorogenic acid, forsythoside B, and obacunone were obtained from the National Institutes for Food and Drug Control (Beijing, China). All standards were determined to have a purity of ≥95% by HPLC. All references were deliquated with methanol to a concentration of 50.0 *μ*g/mL. PTQX granules were produced on a pilot-scale by Jiangyin Tian Jiang Pharmaceutical Co., Ltd. (Jiangsu, China).

DNCB (1-chloro-2,4-dinitrobenzene) and enzyme-linked immunosorbent assay (ELISA) kits for IgE were obtained from Sigma-Aldrich. Mouse Th1/Th2/Th17 and Th17/Treg phenotyping kits were obtained from BD Biosciences (San Jose, CA, USA). All other chemicals and solvents were of the highest commercially available grade.

### 2.2. Chemical Constitution Analysis of PTQX Using Ultra-HPLC- (UHPLC-) LTQ-Orbitrap-Mass Spectrometry (MS)

The Chinese herbal formula PTQX was prepared in TCM granule form using extracts from 9 Chinese medicinal substances, including Rhizoma Atractylodis Macrocephalae (Bai Zhu) 10 g, Radix Pseudostellariae (Tai Zi Shen) 10 g, Rhizoma Dioscoreae (Shan Yao) 15 g, Semen Coicis (Yi Yi Ren) 20 g, Rhizoma Imperatae (Bai Mao Gen) 15 g, Fructus Forsythiae (Lian Qiao) 10 g, Cortex Dictamni (Bai Xian Pi) 10 g, Margarita (Zhen Zhu Feng) 0.3 g, and Radix Glycyrrhizae (Gan Cao) 5 g [10]. This formula has been patented for the treatment of AD (patent no.: ZL2013 1 0328668.4, China).

UHPLC-LTQ-Orbitrap-MS was used to specify the chemical compounds present in PTQX granules. Chromatographic separation was conducted on an Accela UHPLC system (Thermo Fisher Scientific, San Jose, CA, USA). The sample solution was filtered and analysed on a Phenomenex Kinetex C_18_ column (2.1 mm × 100 mm i.d., 1.7 *μ*m). The mobile phase comprised a mixture of 0.1% formic acid (A) and acetonitrile (B). The elution gradient was as follows: 0–20 min, 8–18% B; 20–25 min, 18–40% B; and 25–32 min, 40–85% B. The injection volume was 2 *μ*L, and the flow rate was 300 *μ*L/min. Since flavonoids, phenethyl alcohol glycosides, lignans, saponins, and limonoids were the major structural types mainly detected and identified in the PTQX formula, they might be the main contributions for the AD-related activities. For instance, it has been reported that rutin (compound 16) could be a potential therapeutic agent for the treatment of AD and allergic contact dermatitis [14]. In addition, a previous study showed that isoliquiritigenin (compound 28) has therapeutic effects in the treatment of atopic dermatitis-like skin lesions in mice by inhibiting p38 and ERK activation [15]. Furthermore, glycyrrhizin (compound 29) can also alleviate atopic dermatitis-like symptoms [16].

For the qualitative analysis, an LTQ-Orbitrap XL mass spectrometer was coupled with the LC device via an electrospray ionisation (ESI) interface (Thermo Fisher Scientific, Bremen, Germany). The samples were determined in the negative mode using the following ESI parameters: spray voltage, −3.3 kV; capillary temperature, 325°C; tube lens voltage, −72 V; sheath gas, 45 units; and auxiliary gases, 6 units. The Orbitrap mass analyser was set to the full scan mass range with an *m*/*z* ratio of 110–1100 and resolution of 30,000. The following limits for the potentially expected atoms in the components were set: carbon ≤50, hydrogen ≤80, oxygen ≤30, and nitrogen ≤9. The accuracy error threshold was set at 5 ppm. Xcalibur 2.1 software (Thermo Fisher Scientific) was used to analyse the data. The chemical compounds in PTQX granules were distinguished through comparisons with mass data from the reference standards or fragment information in the literature.

### 2.3. Mouse Model of DNCB-Induced AD

Specific pathogen-free male NC/Nga mice (age: 6–8 weeks) were obtained from the Riken BioResource Centre in Japan. The animals were kept in a room with a controlled temperature of 22 ± 3°C, relative humidity of 55 ± 5%, and illumination with a 12-h light/dark cycle for at least 1 week prior to the experiments. All animals were fed with standard rodent chow daily and fresh tap water ad libitum. All animal experiments were performed under anaesthesia. Ten grams of 2,2,2-tribromoethanol (Avertin, Sigma-Aldrich, USA) was dissolved in 10 ml of tertamyl alcohol to yield a stock solution. A final working solution was prepared by diluting 1 ml of stock solution with 39 ml of isotonic saline. For anaesthesia, the stock solution was administered intraperitoneally (i.p.; 12–15 *μ*l/g of animal body weight). All animal care, use, and experimental procedures were conducted in accordance with the Laboratory Animal Research Committee Guidelines of Guangdong Provincial Hospital of Chinese Medicine (approval number 2016015).

The mice were randomly divided into four experimental groups (*n* = 7-8 per group). AD-like immunological and skin lesions were induced in NC/Nga mice by treatment with DNCB as described previously, with minor modifications [17]. Briefly, DNCB was applied to a patch of dorsal skin, the face, and the back of both ears of each mouse. After the complete removal of dorsal hair from an approximately 8 cm^2^ area, AD-like skin lesions were topically induced by sensitisation with 200 *μ*l of 1% DNCB dissolved in acetone-olive oil (AO, 3 : 1). Additionally, 20-*μ*l aliquots of this 1% DNCB working solution were applied repeatedly to the face and the backs of both ears on days −4 and 0. All mice except those in the control group were challenged with 0.2% DNCB applied thrice weekly for 3 weeks (days 1–20) on the same areas of the skin to induce AD-like lesions. On day 21, the mice were sacrificed under anaesthesia as described above and samples were collected for further analyses. Mice in the control group were treated with vehicle.

**Figure 1:**
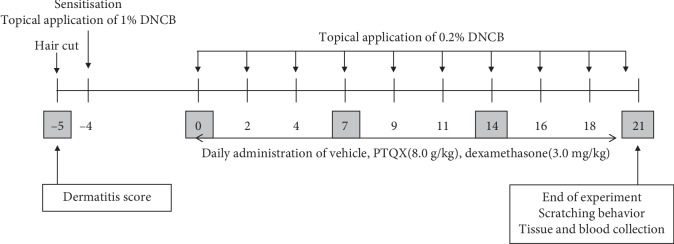
Experimental scheme for the 1-chloro-2,4-dinitrobenzene- (DNCB-) induced model of atopic dermatitis (AD).

For treatment, PTQX was suspended in pure water and administered orally at a volume of 20 ml/kg to the PTQX-treated group (8.0 g/kg) every day since the first sensitisation. A dexamethasone-treated group (10 ml/kg, daily oral 3.0 mg/kg) was maintained as the positive control. The model group received the same volume of pure water. The experimental design is summarised in Figure 1.

### 2.4. Evaluation of Dermatitis Severity and Ear Thickness

After anesthetising mice with 2,2,2-tribromoethanol, the skin lesions were imaged using a digital camera (Canon, Tokyo, Japan) on day 7 of the experiment. We evaluated the dorsal dermatitis scores once weekly according to the previously published standards [17, 18]. The severity of AD was graded on the following scale: 0 = none, 1 = mild, 2 = moderate, and 3 = severe. This scale was applied to each of the four symptoms: (i) erythema/haemorrhage, (ii) oedema, (iii) excoriation/erosion, and (iv) scaling/dryness. The total dermatitis scores can range from 0 to 12 points. We further measured the thicknesses of the right and left auricles weekly during the study period using an electronic digital calliper (Guangxi, China). To minimise technical variation, a single investigator performed all measurements during each experiment.

### 2.5. Evaluation of Scratching Behaviour

NC/Nga mice with AD-like symptoms exhibit pathological and behavioural characteristics very similar to those observed in patients with AD. For elucidation, the duration of scratching behaviour was defined as the time spent rubbing the head and scratching the dorsal skin, nose, and face with the hind limbs. A scratching behaviour event was defined as a single 20-minute incidence of scratching and was measured using a digital camera facing the test box on the penultimate day of the experiment (day 20).

### 2.6. Histology and Immunohistochemistry (IHC)

Histological and immunohistochemical changes can reflect pathological changes in the tissue sections. For comparison, samples of the dorsal skin and one ear were collected from each mouse on the last day of the experiment. The tissues were stored in cold 4% paraformaldehyde overnight and embedded in paraffin wax according to the standard procedures. Subsequently, the tissues were sectioned at a thickness of 4 *μ*m and mounted on silane-coated glass slides. Deparaffinised and rehydrated tissue sections were stained with haematoxylin and eosin (HE) or toluidine blue (TB) to detect infiltrating inflammatory cells or mast cells, respectively. To characterise the infiltrating CD4+ and CD8+ lymphocytes in ear tissues, primary monoclonal rat antibodies against mouse CD8a (clone 53–6.7) and CD4 (GK1.5) were used to indirectly stain the samples. Subsequently, a horseradish peroxidase-(HRP-)conjugated goat anti-mouse IgG-HRP secondary antibody was used to label the bound primary antibodies. Tissue sections were examined using an Olympus BX53 light microscope (Tokyo, Japan).

### 2.7. Flow Cytometry Analysis

Cell suspensions were isolated from the draining lymph nodes of the mice in each group. Suspensions of the mouse spleen and lymph node cells (10 million/ml in media) were activated for 5 hours using phorbol-12-myristate-13-acetate (PMA) and ionomycin (50 ng/ml and 1 *μ*g/ml, respectively) in the presence of BD GolgiStop™ Protein Transport Inhibitor according to the manufacturer's instructions. Flow cytometry was performed on a BD FACSAria™ III device.

### 2.8. Total Serum IgE Levels

At the end of the experiment, blood samples were collected from the mice and serum samples were obtained by centrifugation (3500 rpm for 15 min) and stored at −80°C until use. The total serum IgE levels were measured using a mouse IgE ELISA kit (Sigma-Aldrich) according to the manufacturer's instructions.

### 2.9. Statistical Analyses

Data were expressed as means ± standard errors of the means (SEM). A one-way analysis of variance (ANOVA) or Student's *t*-test was used to determine the significance of differences. *P* values of <0.05 and <0.01 were considered significant.

## 3. Results

### 3.1. Quality Control Analysis of PTQX

**Figure 2:**
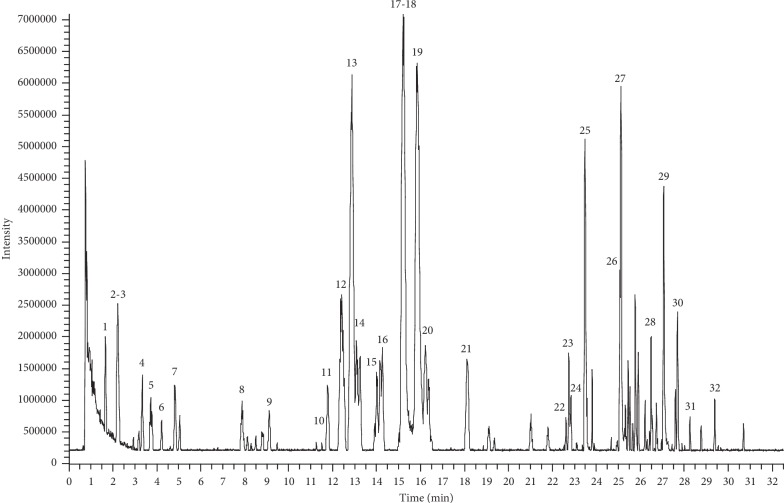
Liquid chromatography- (LC-) mass spectrometry (MS) base peak chromatogram of an extract of PTQX.

The phytochemical constituents in an extract of PTQX granules were identified by UHPLC-LTQ-Orbitrap-MS in the ESI negative-ion mode. A base peak chromatogram of the PTQX granule extract was acquired for structural confirmation (Figure 2). The authentic compounds and their MS/MS fragmentation behaviours were conducted to elucidate the chemical components. Thirty-two components were identified in the PTQX granules through comparisons with the retention and mass behaviours of standards or data in the literature [19–21]. Specifically, the major constituents of the PTQX extract were attributed to Forsythiae Fructus (18 compounds), Glycyrrhizae Radix et Rhizoma (9 compounds), and Dictamni Cortex (4 compounds). The quasimolecular ions of corresponding compounds and their fragment ions in the MS/MS spectra are listed in Table 1.

### 3.2. PTQX Attenuated DNCB-Induced AD-Like Symptoms

**Figure 3:**
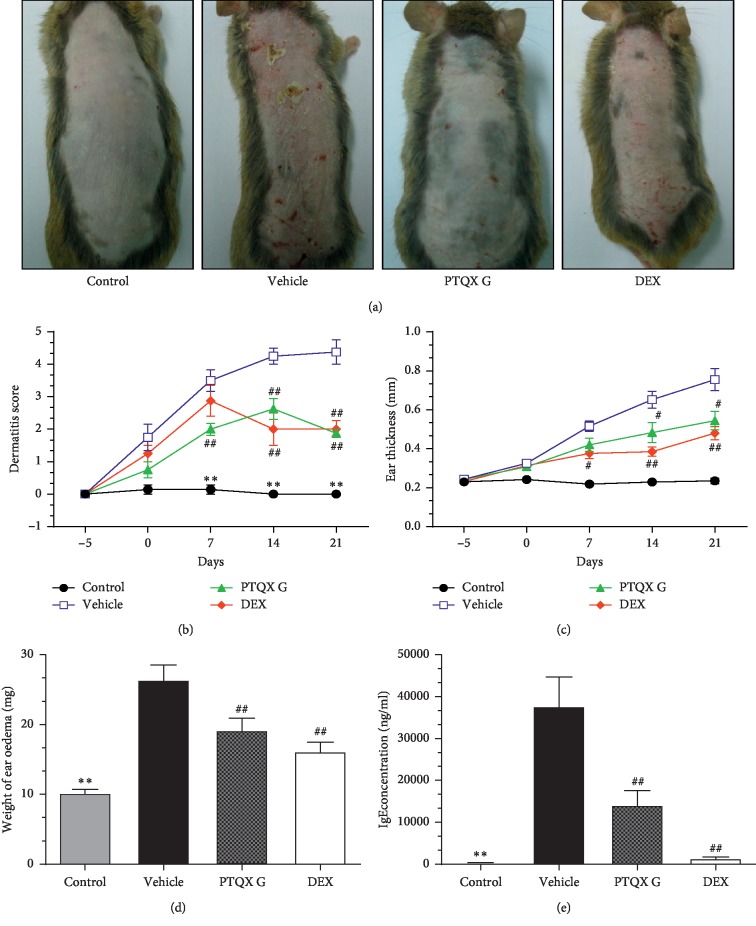
Treatment with PTQX inhibits 1-chloro-2,4-dinitrobenzene- (DNCB-) induced atopic dermatitis- (AD-) like skin inflammation in NC/Nga mice. (a). Representative photographs taken on day 7. (b). Dermatitis scores were evaluated weekly from day −5 to day 21. (c). Ear thickness was measured from day −5 to 21. (d). The effect of PTQX on the ear weights of NC/Nga mice on day 21. (e). PTQX reduces the serum levels of total IgE in NC/Nga mice. The results are expressed as means ± standard errors of the means for 7–8 mice per group. Control, untreated group; vehicle, DNCB-induced group; PTQX, PTQX-treated group; and DEX, dexamethasone-treated group. Statistically significant differences with respect to the control and vehicle groups are expressed as ^*∗*^*P* < 0.05, ^*∗∗*^*P* < 0.01 and ^#^*P* < 0.05, ^##^*P* < 0.01, respectively.

We examined whether PTQX could inhibit DNCB-induced AD-like skin inflammation in mice. Representative dorsal skin photographs, dermatitis scores, and ear thicknesses were obtained from mice treated or not with PTQX to compare AD-like skin lesions. In AD mice, the dorsal skin exhibited severe erythema, erosion, and dryness which was ameliorated by treatment with PTQX (Figure 3(a)). In addition, the dermatitis score was significantly higher in the vehicle-treated group compared with the control group. We further observed that treatment with PTQX or DEX significantly ameliorated the severity of DNCB-induced increases in the dermatitis score (Figure 3(b)). At the end of the experiment (day 21), a measurement of the treated mouse ears revealed that PTQX significantly inhibited the DNCB-induced increase in ear thickness (Figure 3(c)). In other words, the gradual ear oedema induced in NC/Nga mice by DNCB was clearly and similarly inhibited by both DEX and PTQX (Figure 3(d)).

### 3.3. PTQX Decreased Serum IgE Levels

A close correlation has been observed between serum IgE levels and AD. The high IgE levels characteristic of AD place the patient at risk of sensitisation to food allergens and aeroallergens. Therefore, the total serum IgE level is an important indicator in AD. We used an ELISA to detect the total serum IgE levels in the mice from each group. We observed a marked increase in this indicator in the vehicle group, compared with the normal control group (37760 ± 6971 vs. 318.9 ± 59.3 ng/mL). Compared to the vehicle, PTQX significantly inhibited this increase in the total serum IgE level (14145 ± 3457 ng/mL), suggesting that this TCM formula could suppress the IgE synthesis associated with AD. Treatment with DEX also clearly and significantly depressed the increase in total serum IgE levels (Figure 3(e)).

### 3.4. PTQX Inhibits Mast Cell Infiltration and Scratching Frequency in NC/Nga Mice

**Figure 4:**
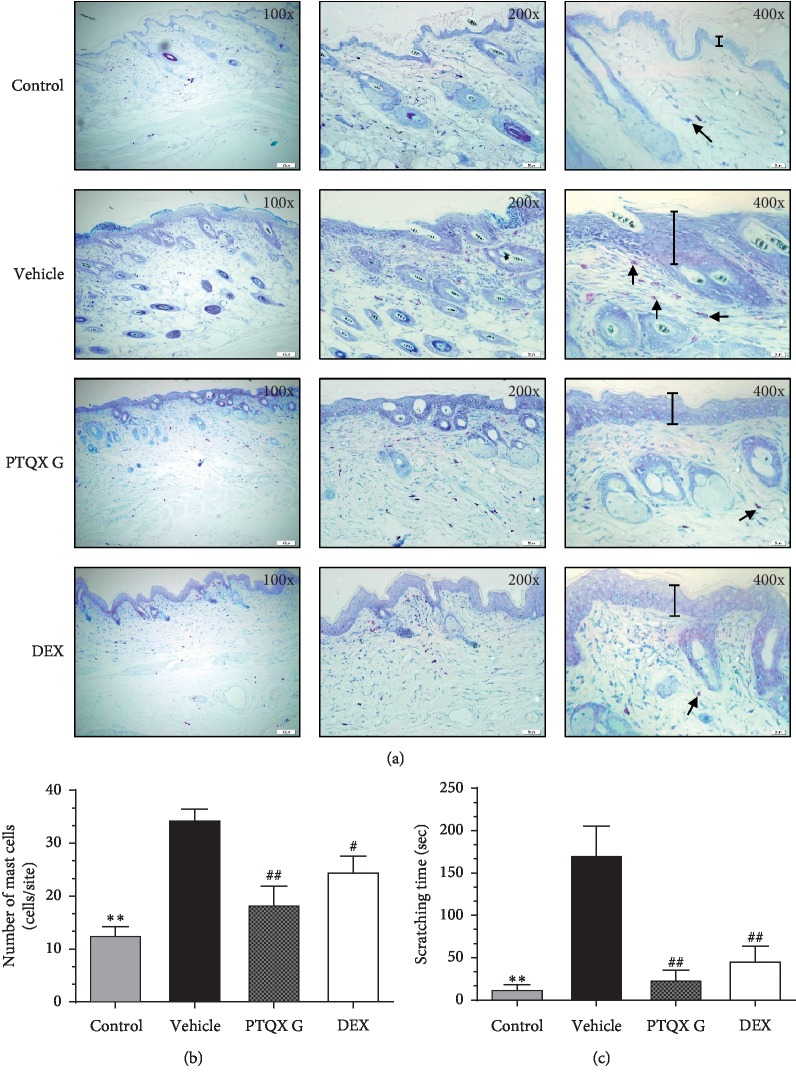
Effect of PTQX on 1-chloro-2,4-dinitrobenzene- (DNCB-) induced mast cell infiltration and scratching frequency in the dermal lesions of NC/Nga mice. (a) Representative images depicting the histological features of skin samples collected on day 21. Toluidine blue (TB) staining was used to identify mast cells (arrows). Cells were counted under a microscope at 100x, 200x, and 400x magnification. (b) Mast cells were counted in five randomly selected sites of TB-stained sections. (c) Effect of PTQX on the DNCB-induced scratching incidence in NC/Nga mice. The scratching time was evaluated on the penultimate day of the experiment (day 20). The results are expressed as the means ± standard errors of the means of 3 mice. Statistically significant differences with respect to the control and vehicle groups are expressed as ^*∗*^*P* < 0.05, ^*∗∗*^*P* < 0.01 and ^#^*P* < 0.05, ^##^*P* < 0.01, respectively.

Mast cells have long been recognised as the major effector cells in the pathogenesis of AD [22, 23]. To determine whether PTQX reduces the infiltration of mast cells into the skin, we stained skin samples with toluidine blue. Larger numbers of mast cells were detected in the dermis of the AD model mice, compared to the normal mice (Figure 4(a)). Sensitisation with DNCB significantly increased the infiltration of mast cells into the dorsal skin lesions (34.5 ± 1.96). Treatment with DEX significantly inhibited this infiltration (24.67 ± 2.843). Similarly, treatment with PTQX also reduced the number of infiltrating mast cells (18.44 ± 3.469) (Figure 4(b)).

The severe itching and scratching associated with AD not only affect a patient's quality of life, but also exacerbate disease progression. Therefore, an analysis of spontaneous scratching behaviour could potentially be used to evaluate the antipruritic efficacy of PTQX in a NC/Nga mouse model of AD [24]. On day 20, we carefully monitored the animals for 20 min and quantified the scratching time, defined as the time spent rubbing their ears, nose, and dorsal skin with their hind paws. DNCB-induced NC/Nga mice (vehicle) exhibited the longest scratching time, 171.2 ± 34.1 s. Comparatively, the PTQX group had a significantly shorter scratching time of 24 ± 11.12 s, an approximately 86% reduction. Treatment with DEX also reduced the DNCB-induced scratching time to 46.25 ± 17.24 s (Figure 4(c)).

### 3.5. PTQX Inhibited DNCB-Induced AD-Like Skin Inflammation in NC/Nga Mice

**Figure 5:**
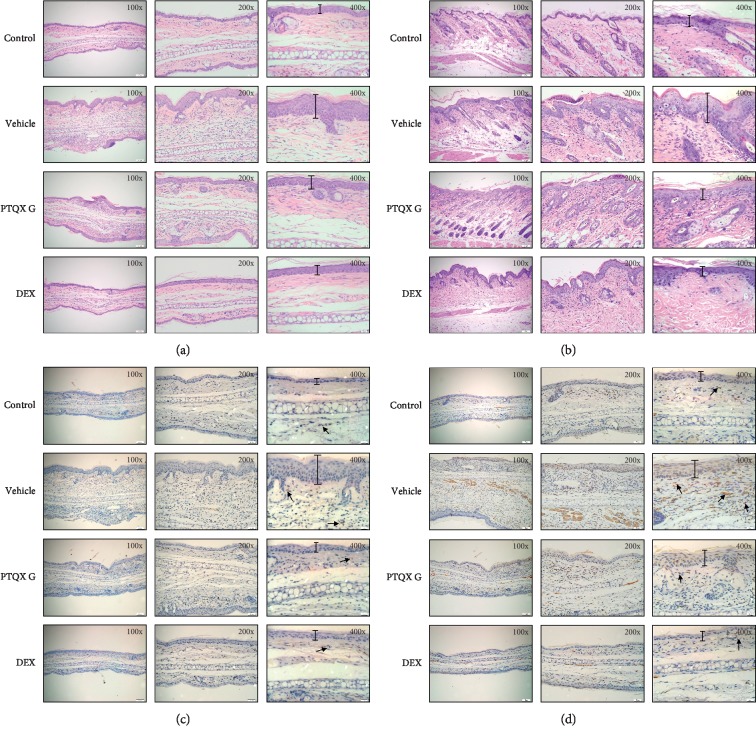
PTQX suppresses tissue inflammation and immune cell infiltration in NC/Nga mice. Atopic dermatitis was induced by the repeated topical application of 1-chloro-2,4-dinitrobenzene (DNCB), with or without PTQX treatment. Sections of the ears (a) and dorsal skin (b) were stained with haematoxylin and eosin (HE). The ear tissues were also subjected to an immunohistochemical analysis of CD4+ (c) and CD8+ (d) T lymphocytes to estimate epidermal inflammation (arrows). A representative example of 3 independent experiments is shown.

To determine whether PTQX could inhibit AD in NC/Nga mice, we used DNCB to induce AD-like skin inflammation in the presence or absence of PTQX. Histologically, PTQX treatment significantly reduced the epidermal thickness, inflammatory cell infiltration, and dense fibrous bundles in the ears of model mice (Figure 5(a)). PTQX treatment also markedly reduced the infiltration of lymphocytes, inflammatory cells, and mononuclear cells into dorsal skin lesions (Figure 5(b)). Furthermore, IHC revealed that the ears of mice with DNCB-induced swelling were more strongly stained with CD4- and CD8-specific antibodies, compared to the vehicle mice. The populations of CD8+ and CD4+ T cells in the ears also decreased significantly after treatment with PTQX (Figure 5(c) and Figure 5(d)), confirming that this formula attenuated the cutaneous inflammation induced by DNCB in NC/Nga mice.

### 3.6. PTQX Regulates CD4+ T-Cell Proliferation and Differentiation

**Figure 6:**
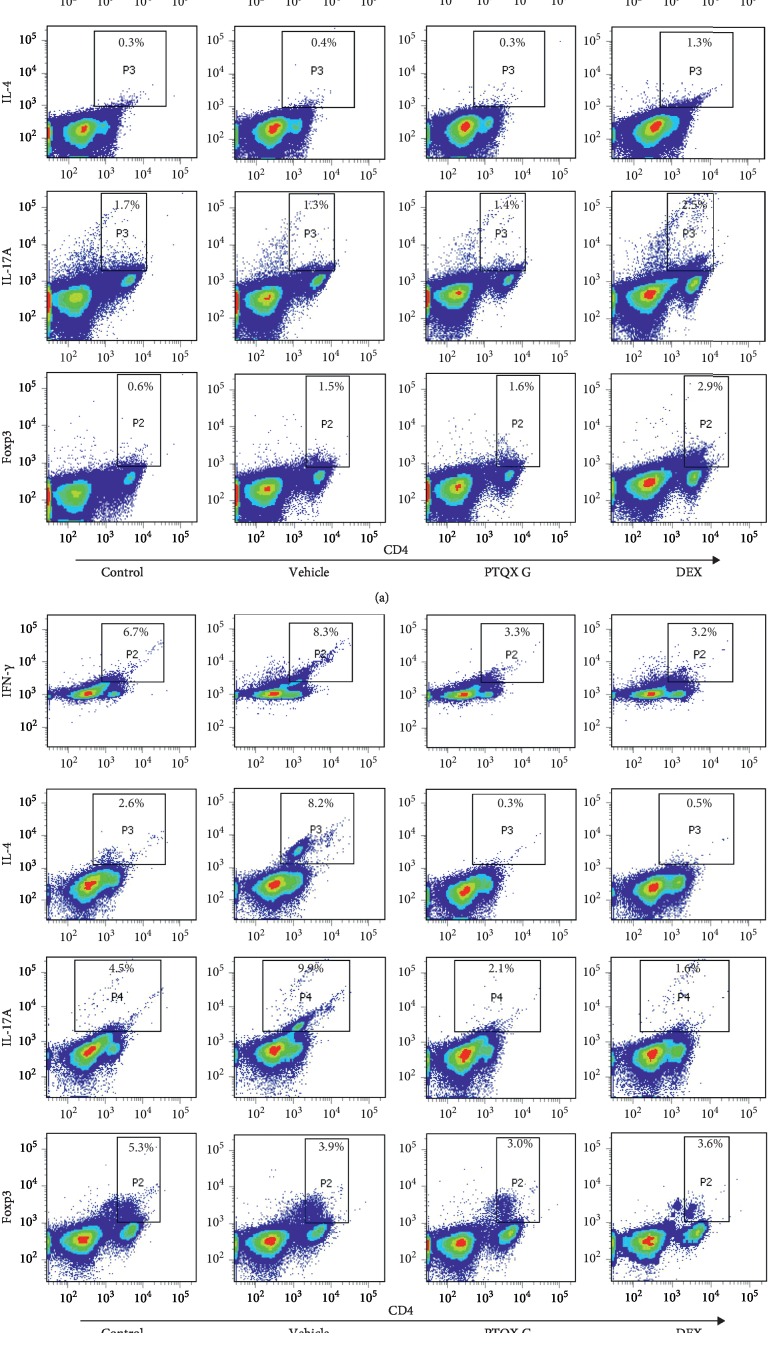
Treatment with PTQX inhibits the differentiation of T-helper 2 (Th2) and Th17 cells in the draining lymph nodes of NC/Nga mice with 1-chloro-2,4-dinitrobenzene- (DNCB-) induced atopic dermatitis. CD4+ Th1, Th2, Th17, and regulatory T (Treg) lymphocyte populations were selected by costaining for IFN-*γ*, IL-4, IL-17A, and Foxp3, respectively. Flow cytometry was used to evaluate these populations of lymphocytes in the spleens (a) and lymph nodes (b) of mice. A representative example of 2 replicate independent experiments is shown.

Our interest in and understanding of the importance of CD4+ T cells in the development of AD have increased in recent years [25]. Upon activation, CD4+ T cells mainly differentiate into four subsets: Th1, Th2, Th17, and Treg. Studies of Th1/Th2 and Th17/Treg differentiation patterns during the progression of AD indicate the importance of CD4+ cells in this disease [5, 26]. Therefore, we focused on the effect of PTQX on the contributions of CD4+ T cells to immunomodulation in AD. Interestingly, our results showed that PTQX had almost no effect on CD4+ T-cell differentiation in the spleen (Figure 6(a)). In the lymph nodes, however, PTQX significantly inhibited the proliferation of CD4+ IL-4 and CD4+ Foxp3 Treg cells, compared to vehicle treatment (Figure 6(b)). Our study partly explains the mechanism underlying the pharmacological effects of PTQX. Specifically, this formula regulates the Th17/Treg balance to inhibit the development of AD. Our results indicate that PTQX suppressed the symptoms of DNCB-induced AD by modulating CD4+ T-cell proliferation and differentiation and particularly by regulating the Th17/Treg balance.

## 4. Discussion

Various studies have long demonstrated the clinical effects of TCM formulas and related treatments for AD [27]. In TCM, Si Wan Feng described a condition comparable to atopic eczema/AD and the treatment of this disease requires the differentiation of the chief syndrome and an analysis and determination of treatment strategies. In TCM, treatment aims to strengthen the body against disease and strongly emphasises Qi (i.e., vital energy) and the Yin-Yang balance (i.e., negative and positive equilibrium) as factors contributing to health and well-being. According to TCM theory, the aetiology of AD is closely related to “wind,” “dampness,” and “heat” [28] and traditional formulas can cleanse and clear these factors. Therefore, TCM is intended to improve a person's general health and wellness while treating specific diseases or common ailments. However, more high-quality RCTs are needed to assess the efficacy of TCM. The Chinese herbal formula PTQX is intended to simultaneously invigorate the spleen while reducing dampness, clearing heart-fire, and relieving pruritus.

In 2015, we reported the results of an assessor-blind, multicentre RCT of PTQX for the treatment of AD [9, 29]. Still, the molecular mechanism underlying the effects of PTQX against AD urgently requires attention. In this study, we found that PTQX could improve the AD-like symptoms of redness, eczema, and pruritus induced by DNCB in NC/Nga mice. Our previous experimental and clinical study also demonstrated that PTQX was an effective treatment for AD. Therefore, it would be helpful to further clarify the mechanism of action of PTQX.

To date, several murine models of AD have been established, including transgenic, NC/Nga, NOA, C57BL/6, and BALB/c mice [30–33]. Although previous studies have used animal models displaying some of the characteristic histopathological features of AD, the disease pathogenesis and mechanisms remain incompletely understood. This disease is characterised by the infiltration of macrophage and inflammatory cells and Langerhans cells into the dermis, activation of T lymphocytes and dendritic cells (DCs), and the proliferation and activation of mast cells [34]. Our present study, based on NC/Nga AD model mice, provides important information about the causes of the basic and clinical AD-like symptoms induced by DNCB. Mast cells are critical to the induction of allergic diseases such as asthma, allergic rhinitis, and AD [35]. Here, our findings suggest that PTQX suppresses the inflammatory infiltration of mast cells, which are thought to contribute to the pathogenesis of AD. Our results indicate that PTQX can reduce the infiltration of mast cells into skin lesions and alleviate the symptoms of AD.

We further found that PTQX could inhibit the increases in epidermal and dermal thickness and serum IgE production observed in AD model mice. We were particularly interested in the immune regulatory effects of PTQX on the balance between the Th1, Th2, Th17, and Treg subtypes in the spleen and lymph nodes. Recent research has suggested that the additional activation of the Th22-, Th17/IL-23-, and Th1-related cytokine pathways depends on the subtype of the disease [36], while Th2 cells have been implicated in AD pathogenesis and development [37]. A skewed Th17/Treg balance may also play a vital role in several autoimmune, inflammatory, and allergic reactions. Nevertheless, the possible contribution of the Th17/Treg imbalance to AD remains unclear and further studies are needed. Our results suggest that PTQX treatment reduced the populations of Th1 and Th2 cells and regulated the ratio of Th17/Treg cells in DNCB-treated NC/Nga mice. Accordingly, treatment with PTQX may improve the Th17/Treg immune balance and help to regulate inflammation.

## 5. Conclusion

Currently, AD cannot be fully cured or controlled. Our previous clinical studies have shown that PTQX can effectively reduce the disease severity and improve the quality of life and self-assessment of patients with moderate-to-severe AD. This study further shows that therapeutic effects of PTQX on AD by regulating Th2/Th17 cell balance and suppressing mast cell infiltration while effectively relieving pruritis and reducing the inflammatory response in a NC/Nga mouse model. Our results suggest that PTQX might be a good candidate for the treatment of AD.

## Figures and Tables

**Table 1:** Compounds detected and identified in PTQX formula.

No.	*t*_R_ (min)	[M − H]^−^or[M + COO^−^]^−^(ppm)	Identification	Formula	Resource
1.	1.66	375.12930 (0.73)	Adoxosidic acid	C_16_H_24_O_10_	Ff
2.	2.16	353.08701 (0.30)	Neochlorogenic acid	C_16_H_18_O_9_	Ir, Am, Ff
3.	2.23	461.16556 (0.21)	Forsythoside E	C_20_H_30_O_12_	Ff
4.	3.34	209.04535 (0.35)	Unknown	C_10_H_10_O_5_	Grr
5.	3.71	353.08701 (0.39)	Chlorogenic acid^b^	C_16_H_18_O_9_	Ir, Am, Ff
6.	4.22	353.08682 (0.11)	Cryptochlorogenic acid	C_16_H_18_O_9_	Ir, Am, Ff
7.	4.80	179.03476 (−0.46)	Caffeic acid	C_9_H_8_O_4_	Ff, Ir
8.	7.85	229.10760 (0.55)	Unknown	C_11_H_18_O_5_	Dc
9.	9.11	495.14914 (−0.56)	Forsythenside A^b^	C_22_H_26_O_10_	Ff
10.	11.25	581.18518^a^ (−1.30)	Forsythiaside B	C_26_H_32_O_12_	Ff
11.	11.76	609.18066 (−0.74)	Calceolarioside C/lianqiaoxinoside C	C_28_H_34_O_15_	Ff
12.	12.42	417.11813 (0.12)	Liquiritin^b^	C_21_H_22_O_9_	Grr
13.	12.86	623.19684 (−0.21)	Isoforsythiaside A^b^	C_29_H_36_O_15_	Ff
14.	13.08	549.16028 (0.01)	Licuroside	C_26_H_30_O_13_	Grr
15.	14.01	609.18097 (−0.98)	Calceolarioside C/lianqiaoxinoside C	C_28_H_34_O_15_	Ff
16.	14.17	609.14447(−1.09)	Rutin	C_27_H_30_O_16_	Ff
17.	15.01	527.21161^a^ (−0.69)	Forsythenside G	C_24_H_34_O_10_	Ff
18.	15.21	623.19702(−0.03)	Forsythoside A^b^	C_29_H_36_O_15_	Ff
19.	15.70	755.23737(−1.94)	Forsythoside B^b^	C_34_H_44_O_19_	Ff
20.	16.19	519.18616 (−0.48)	Epipinoresinol-4-*O-β*-D-glucopyranoside	C_36_H_32_O_11_	Ff
21.	18.11	565.19086(-1.26)	Pinoresinol-4-*O-β*-D-glucopyranoside	C_36_H_32_O_11_	Ff
12.	21.79	475.12305^a^ (0.5)	Ononin	C_22_H_22_O_9_	Grr
23.	22.83	417.11844 (0.43)	Isoliquiritin^b^	C_21_H_22_O_9_	Grr
24.	23.01	255.06586 (0.68)	Liquirtigenin^b^	C_15_H_12_O_4_	Grr
25.	23.46	579.20715^a^ (−0.07)	Forsythin^b^	C_27_H_34_O_11_	Ff
26.	25.05	1011.42535 (−0.73)	Unknown	C_70_H_60_O_7_	Grr
27.	25.10	849.37482 (−0.80)	Unknown	C_39_H_62_O_20_	Grr
28.	26.73	255.06592 (0.74)	Isoliquiritigenin^b^	C_15_H_12_O_4_	Grr
29.	27.06	821.39496 (−0.45)	Glycyrrhizin^b^	C_42_H_62_O_16_	Grr
30.	27.67	515.19080^a^ (−0.38)	Limonin	C_26_H_30_O_8_	Dc
31.	28.26	483.16458 (−0.93)	Limonin diosphenol	C_26_H_28_O_9_	Dc
32.	30.70	453.19064 (−0.69)	Obacunone^b^	C_26_H_30_O_7_	Dc

^a^[M + HCOO]-; ^b^compared with the standard compound. Ff: Forsythiae Fructus; Grr: Glycyrrhizae Radix et Rhizoma; Dc: Dictamni Cortex; Ir: Imperatae Rhizoma; Am: Atractylodis Macrocephalae; Dr: Dioscoreae Rhizoma; Pr: Pseudostellariae Radix; Po: Poria.

## Data Availability

The data used to support the findings of this study are included within the article.

## Abbreviations

AD: Atopic dermatitis
AO: Acetone-olive oil
DCs: Dendritic cells
DEX: Dexamethasone
DNCB: 1-Chloro-2,4-dinitrobenzene
ELISA: Enzyme-linked immunosorbent assay
HE: Haematoxylin and eosin
HPLC: High-performance liquid chromatography
ESI: Electrospray ionisation
IgE: Immunoglobulin E
IHC: Immunohistochemistry
PMA: Phorbol-12-myristate-13-acetate
PTQX: Chinese herbal formula Pei Tu Qing Xin
QOL: Quality of life
SEM: Standard error of mean
TCM: Traditional Chinese medicine
TB: Toluidine blue
Th: T-helper cell type
Treg: Regulatory T cell.
